# Options to Determine Pathological Response of Axillary Lymph Node Metastasis after Neoadjuvant Chemotherapy in Advanced Breast Cancer

**DOI:** 10.3390/cancers13164167

**Published:** 2021-08-19

**Authors:** Vijayashree Murthy, Jessica Young, Yoshihisa Tokumaru, Marie Quinn, Stephen B. Edge, Kazuaki Takabe

**Affiliations:** 1 Breast Surgery, Department of Surgical Oncology, Roswell Park Comprehensive Cancer Center, Buffalo, NY 14263, USA; drvijumurthy@gmail.com (V.M.); jessica.young@roswellpark.org (J.Y.); Yoshihisa.Tokumaru@roswellpark.org (Y.T.); Stephen.edge@roswellpark.org (S.B.E.); 2 Department of Radiology, Roswell Park Comprehensive Cancer Center, Buffalo, NY 14263, USA; marie.quinn@roswellpark.org; 3 Department of Surgery, Jacobs School of Medicine and Biomedical Sciences, State University of New York, Buffalo, NY 14263, USA; 4 Department of Gastrointestinal Tract Surgery, Fukushima Medical University School of Medicine, Fukushima 960-1295, Japan; 5 Department of Gastroenterological Surgery, Yokohama City University Graduate School of Medicine, Yokohama 236-0004, Japan; 6 Department of Surgery, Niigata University Graduate School of Medical and Dental Sciences, Niigata 951-8510, Japan; 7 Department of Breast Surgery and Oncology, Tokyo Medical University, Tokyo 160-8402, Japan

**Keywords:** breast cancer, lymph node, neoadjuvant therapy, neoadjuvant chemotherapy, sentinel lymph node biopsy, locally advanced breast cancer, positive lymph node, pCR, pathological complete response, surgical de-escalation

## Abstract

**Simple Summary:**

Neoadjuvant therapy instituted prior to definitive surgery helps to reduce the tumor burden in the breast and axilla. De-escalation of surgery in the axilla may allow removal of just the involved nodes and sentinel nodes for determination of pathological response of previously biopsy proven positive axillary nodes. In order to attain the optimal surgical results with minimum risk of complications, it is important to choose the accurate method of identification of these positive nodes. In this review, we examine the different options to assure identification of the nodes deemed positive before neoadjuvant therapy, at the time of definitive surgery.

**Abstract:**

Increasing use of neoadjuvant therapy in large tumors or node positive disease in breast cancer patients or hormone negative and HER 2 overexpressing cancers often gives rise to complete clinical response, with resolution of disease in the breast and axilla. These results have raised important questions to deescalate loco-regional surgical treatment options with minimum recurrence risk and treatment related morbidity. Although there is excellent prognosis following clinical response, the primary goal of surgery still remains to confirm complete pathological response in the biopsied node that was previously positive and now clinically/radiologically negative (ycN0). Biopsied lymph nodes are often marked with a clip to allow future identification at the time of definitive surgery. The goal of lymph node surgery in oncology is that it should be accurate, hence the significance of localizing the biopsied node. This article aims to review the different options to localize the deemed positive node at the time of definitive surgery, in order to help determine pathological response after neoadjuvant therapy.

## 1. Introduction

Neoadjuvant systemic therapy specifically neoadjuvant chemotherapy (NAC) has been traditionally used for locally advanced (Clinical T3N1-N3M0) and inflammatory breast cancer (T4d-N±) to make the tumor more operable. However recently, the indications have been extended to operable hormone negative and HER2 positive cancers given better responses. Notably 40–50% of patients have a complete response in the nodes following NAC [1,2]. Complete nodal response is more likely to be achieved in tumors with high nuclear grade and in hormone receptor negative and HER2 positive tumors [2]. Hormone positive breast cancer is less sensitive to NAC than HER2 positive or triple negative breast cancers (TNBC) [3,4]. Distinct advantages of neoadjuvant systemic therapy include not only reducing the extent of surgery on the breast (mastectomy or breast conservation) and assessing an in vivo response to the treatment, but also reduction of tumor volume in the regional nodes [5]. Currently surgical management of axillary lymph nodes after NAC includes axillary lymph node dissection (ALND), dual tracer sentinel lymph node biopsy (SLNB) with or without specific marking of previously positive lymph node, and targeted axillary lymph node dissection (TAD). To date, there is no evidence to support the choice of operation by specific subtype of breast cancer although the response to NAC significantly differs by subtypes.

NSABP B-27 was a landmark trial that demonstrated that SLNB was not inferior to ALND as surgical management following NAC [6]. It compared doxorubicin-cyclophosphamide with pre-op and post-op docetaxel and showed a significant increase in pathological complete response (pCR) in the pre-op docetaxel arm (26% vs. 13%; *p* < 0.001) but no difference in disease free survival (DFS) or overall survival (OS). Some of the patients underwent sentinel lymph node (SLN) removal prior to the standard axillary dissection. 84.8% of them had successful sentinel node removal, and the false negative rate was 10.7%. Four additional landmark trials that prospectively addressed this issue included the American College of Surgeons Oncology Group (ACOSOG) Z1071, GANEA 2, SENTINA (Sentinel Neoadjuvant) and the SN-FNAC trial [6,7,8,9,10] (Table 1). Patients who presented with complete clinical axillary response following NAC were subjected to SLNB and ALND, with false negative rate (FNR) ranging from 8.4% (in SN FNAC trial) to 14.2% (in SENTINA trial). All these trials had a pre-specified threshold cut off of FNR to be 10%. One of the more important findings of these trials was that after NAC, SLN metastases of any size were significant and therefore should be considered positive. Some authors even promote the mandatory use of LN examination by immunohistochemistry (IHC) [9]. Additionally, using dual tracers for SLN identification yielded more sentinel nodes and lowered the FNR. Thus, the more morbid complete ALND is being potentially avoided in a large number of node positive patients if the nodes can be accurately proven negative by more limited node surgery after NAC [11,12,13].

Post-NAC axillary staging poses unique challenges in identification of previously positive node(s) in the axilla. Some of the reasons quoted are failure to observe blue dye alone in the node or failure to map the axilla due to treatment-related fibrosis. Presently the adopted standard method of axillary LN staging after NAC in breast cancer is SLNB using a dual tracer method with radioisotope lymphoscintigraphy with technetium labelled colloid and injection of a blue dye (isosulfan or methylene). Based on the findings of the abovementioned trials, retrieving only one or two sentinel lymph nodes is associated with a high FNR (24.3% for one SLN vs. 18.5% and 12.6% for 2 SLNs removed) [7,9] and there is no role for blind node sampling or harvesting. In a metanalysis of 13 studies including 1921 patients, Tee et al. found the pooled IR was 90% with FNR being 19% with single method of mapping vs. 11% with dual mapping. FNR was observed to be 20% with one SLN removed and 12 and 4% with two and three SLN removed respectively [14].

Staging of the axilla with biopsy of suspicious nodes has become more common practice in patients with newly diagnosed breast cancer prior to surgery or neoadjuvant treatment. MRI and sonographic evaluation of the axilla has been incorporated into the imaging workup at many centers. Marking of biopsied axillary lymph nodes (ALNs) with metallic markers, similar to what is done for suspicious breast lesions, is being adopted in clinical practice [7]. In as many as one third of cases, the node that was positive before NAC is not mapped by SLNB. To assure this node (s) is identified and removed to assess response, it is necessary to mark this node. This is usually done with a radio opaque clip placed at the time of biopsy [15]. National Comprehensive Cancer Network (NCCN) guidelines for patients with node positive disease prior to NAC, recommends marking the positive biopsied lymph nodes in some form (by ink/clip/marker) and their subsequent removal, and removing at least 3 sentinel nodes using the dual tracer method, to reduce the FNR of SLNB to <10% when performed after NAC [16]. When multiple axillary lymph nodes are suspicious for metastasis, NCCN guideline recommends accurate diagnosis and multiple clip placements. However, there is no clear mention on an upper limit to the number of nodes to be marked. Once lymph node involvement with metastasis is documented, surgical removal of the biopsy proven positive lymph node is important. The concept of targeted axillary lymph node dissection (TAD) has recently been introduced, which involves removal of a node with documented metastases (based on pre-treatment biopsy and marking) in addition to removal of other sentinel nodes most likely to harbor disease. ALND is indicated after NAC when residual LN metastasis is detected by clinical physical examination and/or imaging based on NCCN Guidelines 2021. SLNB with selective LN removal or targeted axillary dissection may be applied to the patients who responded to NAC to the point that its clinical assessment is negative (cN0). Further, it is important to note that radiation to the axillary region should follow SLNB for local control unless ALND was performed. This is because residual tumor cells may be present in the regional lymph node area [16]. In a recent systematic review, Swarnkar et al. emphasize the need for optimizing the FNR in patients receiving NAC for a biopsy-proven node-positive breast, and state that under staging the residual axillary disease can potentially result in undertreatment of adjuvant systemic therapy and compromise of oncological outcome in patients particularly with HER2-positive and TNBC where residual disease is used to guide the use of capecitabine (for TNBC) and TDM-1 (for HER2 positive) [17].

While the placement of a clip in the tumor in the breast prior to NAC is standard to facilitate localization of the tumor site for surgery, similar localization of axillary LNs is more challenging and there is no standard for marking. Considering the widespread use and convenience of marking the breast tumor site with a clip, this was organically the first technique tested for axillary LNs as well as first seen in the ACOSOG Z1071 trial [7]. During SLNB, specimen radiograph of the SLNs can show the clip inside, or the pathologist can identify it in the node. Theoretically, the previously positive biopsied node should be one of the sentinel nodes. However, this is not always the case. Having to find the clipped lymph node presents issues. The clipped node cannot be directly visualized by the surgeon on table and needs the use of intraoperative ultrasound or radiography. The size of the clip is so small that it is seldom easily visualized using the C-arm. At times, it has been found to be extruded on to the operating table or drapes. Failure to visualize or retrieve the clips leads to removal of more lymph nodes and at times to a full axillary node dissection (and its associated morbidity) and the addition of extra radiation due to fluoroscopy for additional localization. Given the importance of accurate assessment of the axilla and minimization of morbidity of extensive axillary surgery, surgeons must have a better approach to access the targeted nodes in the axilla, while performing SLNB after NAC. Many techniques for TAD have been described and have mostly been an adaptation of techniques used for localization of non- palpable lesions in the breast and these include tattoo with charcoal or carbon, wire localization, ultrasound visible clip, magnetic seed, radioactive iodine I^125^ seed placement and radiofrequency identification devices. This article seeks to critically discuss each of these methods in detail including their benefits and drawbacks when performing localization of the clipped node in the axilla (Table 2).

## 2. Techniques to Optimize Removal of Targeted or Clipped Positive Node

### 2.1. Carbon Suspension/Charcoal Tattoo

Black carbon dye or charcoal is an alternative method to localize the axillary node at biopsy prior to any form of neoadjuvant treatment in N+ axilla. The concept of tattooing tumor has been based on the experience in the gastrointestinal tract wherein tattooing is widely used for marking lesions or tumors biopsied during endoscopy. India ink tattoos of colonic lesions remain identifiable over a long period of time [18].

**Figure 1:**
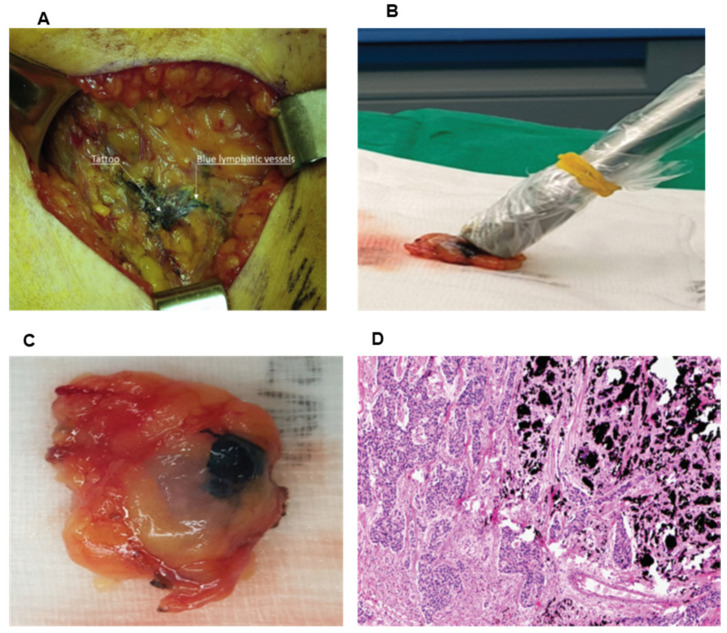
Affected lymph node tattooed with carbon black and sentinel lymph node (**A**) Intraoperative picture of surgical field of axilla- black pigment and blue dye overlap in large areas but blue dyed efferent lymphatic vessels demonstrate the presence of sentinel node. (**B**) Handheld probe confirmation of the “hot” sentinel node. (**C**) Macroscopic appearance of the tattooed lymph node. (**D**) Microscopic findings of tattooed lymph node with extended areas of black pigment granules in the cortex (Hematoxylin and eosin staining, magnification ×25). From Park et al. [21]. Feasibility of Charcoal Tattooing of Cytology-Proven Metastatic Axillary Lymph Node at Diagnosis and Sentinel Lymph Node Biopsy after Neoadjuvant Chemotherapy in Breast Cancer Patients. *Cancer Res. Treat.* **2018**, *50*, 801–812. (Copyright obtained from the Creative Commons under license NC 4.0).

The method of carbon localization was first described to delineate non-palpable breast lesions as early as 1983 by Svane [19], wherein using a stereotactic biopsy instrument, an aqueous suspension of carbon particles was injected so as to leave a distinct trail from the lesion to the skin, and in their experience the authors found that the carbon does not diffuse into the surrounding tissues and hence the marking could be done well in advance of surgery. This concept has now more recently been adopted for axillary nodes. Typically, ultrasound-guided fine needle aspiration or core needle biopsy of the axillary node is done followed by injection of 0.1 to 0.5 mL of a sterile black suspension Spot^TM^ (GI Supply, Mechanicsburg, PA, USA) ink into the cortex of the sampled node and adjacent soft tissue. Spot^TM^ is the first FDA-approved product for tattooing of tissue and contains a suspension containingwater, glycerol, polysorbate 80, benzyl alcohol, simethicone and high purity carbon black. At the time of definitive surgery, SLN mapping is performed in all patients with peritumoral injection of 4–5 mL of isosulfan blue dye and/or 1 or 2 mCi of periareolar Tc-sulfur colloid the same day or day before surgery, respectively. The black, tattooed, perinodal fat guides the surgeon to the targeted tattooed node (Figure 1). The identified black nodes are then examined to determine whether the node is blue, ‘‘hot’’or firm on palpation and sent for frozen section. Studies that have used this technique have looked at identification of black pigment and concordance between sentinel and tattooed nodes. In a study of 28 patients and 36 lymph node specimens Choy et al. used a carbon suspension (Spot^TM^) to mark positive ALNs. Sixteen patients underwent immediate surgery and 12 patients had surgery after NAC [20]. The average number of days between tattooing and surgery was 22.9 days (range 1–62 days) for surgery first group, and 130 days (range 74–211 days) for NAC first group. Overall identification rate (IR) of tattooed ALNs was 96.5%, with correlation between tattooed nodes and SLNs at 96.4%. Park et al. used an activated charcoal suspension (Charcotrace; Healthdirect, Haymarket, New South Wales, Australia) in 20 patients before NAC [21]. Intraoperative IR was 100%, and correlation between tattooed nodes and SLNs was 75%, and were able to identify all those nodes intraoperatively up to 197 days later. In a multiinstitution study of 63 patients with node positive disease prior to NAC, SpotTM (Endoscopic marker, GI Supply, Camp Hill, PA, USA) ink was injected into the cortex of the biopsied node. Intraoperative IR was 95% and correlation of tattooed and SLN was 80% [22]. In another study of 76 women (19 with surgery first and 57 with NAC) using carbon suspension Spot TM, Patel et al. found that the mean time from tattoo to surgery was 21 days (range 1–62 days) for surgery first and 148 (range 71–257) days for NAC patients. IR was 100% in NAC group with a high concordance (98.5%) between tattooed node and SLN, perhaps due to higher technical efficiency as compared to the previous studies [23].

The cost of the ink is $100 per 5 mL vial. An advantage is that injection of tattoo can be easily performed under ultrasound guidance and is well tolerated by patients. The tattoo technique provides an easy method to verify the accuracy of preoperative pathological evaluation of nodes, requiring no additional imaging to either ascertain the location of the node or localization procedure prior to definitive surgery, thus decreasing cost and improving convenience. Above all, the tattoo technique does not interfere with a standard SLNB nor histopathological evaluation of the nodes and allows for correlation to pretreatment-biopsied lymph nodes, making it an attractive technique and one that is readily exportable to low resource environments.

Limitations to this technique include operator and site of injection variability. The black ink identified intraoperatively has sometimes not been identified histologically. This may be due to the fact that during processing, adipose tissue containing tattoo ink is dissected away from the surrounding lymph node and not captured on final histology or when the focus of the black pigment is too small, it may fail to be captured within the level sampled histologically. But intraop identification of tattoo may be sufficicent without histological confirmation. Although the distinction between the blue dye and black ink can be subtle, the tattoo ink displays a black greyish hue, while the isosulfan blue is an intense peacock blue, this ambiguity can be resolved by tracing the blue marking of lymphatics as well as reliance on radiotracer signaling. One study has reported foreign body granuloma in 3% cases [24].

### 2.2. Wire Localization

**Figure 2:**
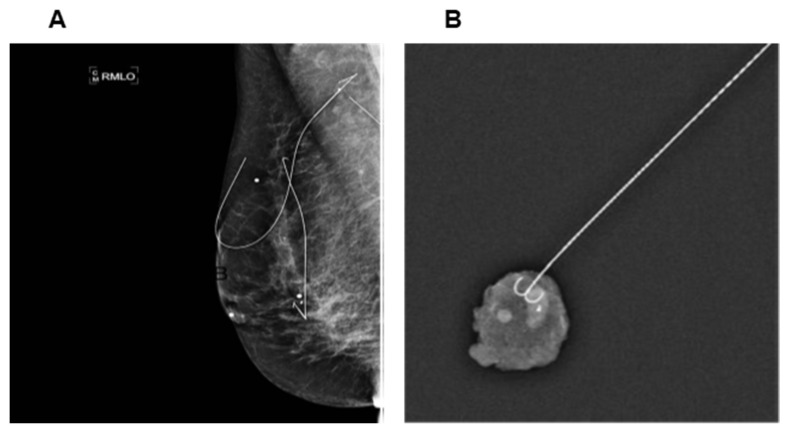
Wire localization. (**A**) Mammogram with wire localized to the positive axillary lymph node and breast lesion. (**B**) Specimen radiograph of wire localization of the clipped node. From Balasubramanian et al. [25]. Wire guided localisation for targeted axillary node dissection is accurate in axillary staging in node positive breast cancer following neoadjuvant chemotherapy. *Eur. J. Surg. Oncol.* **2020**, *46*, 1028–1033 (Copyright obtained from Rightslink Copyright Clearance Center under license number 4910321504420).

Placement of a thin metal wire to localize non-palpable breast lesions prior to definitive surgery has been applied for many decades and since been applied to axillary nodes as well. Wires adapted for target localization are deployed via ultrasound guidance and are available in two forms- fixed and repositionable. These wires are typically loaded on a 7 cm 20-gauge needle with a 20 cm beaded breast localization wire (Figure 2). Fixed wires are available with a variety of tip configurations (such as spring hook wire−Kopans breast lesion localization needle, X shaped hook wire−XReidy breast lesion localization needle, Q shaped wire−Q wire system) to anchor in the target, while repositionable wires have an anchoring tip that can be retracted back to the delivery system if it needs to be repositioned (such as J curve wire- Homer breast localization needle, side barb wire−Hawkins I and II breast localization needle).

In the first reported series of 107 patients from the University Hospitals of Cleveland, the biopsy clip was retrieved in 97.3% of patients with wire localization with no reported complications. This was significantly higher than 79.4% of patients who did not have wire localization (*p* = 0.0043) [26]. In a study of 25 patients at the Guys and St Thomas Hospital, UK in 2017, US guided wire placement was done on the day prior or the day of surgery [25]. The median number of nodes retrieved was 3 (range 1–5). Twenty-three of the clipped nodes were identified either during intraoperative radiography or final histopathology, resulting in clipped node IR of 92%. In a German study of 30 patients with T1-T3 invasive breast cancer with node positive disease and pre-NAC clip placement, 24 out of 30 patients underwent flexible marking wire localization (ultrasound guided in 20 and mammogram guided in 4). The IR for targeted lymph node was 70.8% (17/24 cases) [27]. The authors reported that this was the first prospective evaluation of the feasibility of ultrasound -guided wire localization of a clipped target LN after NAC, and the practical challenge for the surgeons was the displacement of the wire due to patient movement after insertion or surgeon manipulation in the soft fatty axillary tissue during preparation, as also migration of the wire from the clipped node to the surrounding axillary tissue. They concluded that ultrasound (US)-guided wire localization of the clipped axillary node was not appropriate for routine clinical use, especially those that involve additional invasive localization procedures.

As computed tomography (CT)-guided wire localizations have been previously used for pulmonary lesions, Trinh et al. extrapolated the same concept for localization of axillary nodes (marked with clips prior to NAC). This was mainly to evaluate the feasibility of CT-guided wire localization where US localization was unsuccessful. In a small study of five patients, using the needle/hook wire system the node was localized and a post- procedure CT was performed to show the final position of the wire before the patient was taken to surgery [28]. The procedure time was 21–38 min with no complications and identification of the clipped node was seen in 4/5 cases (IR 80%). García-Novoa et al. performed wire localization of clipped axillary nodes in 42 patients and found the IR to be 100% and a high correlation between the wire marked lymph node and the SLN (80%) and with no complications [13]. More recently, Alarcón and colleagues performed SLNB and removal of clipped positive node after NAC using ultrasound guided wire hook placement 30 minutes prior to the definitive surgery in 28 of 103 patients, and found the IR of the clipped node to be 100% with FNR of 0% with no migration or complications. The authors concluded that wire placement immediately prior to surgery and an accurate surgical technique during the nodal excision minimize the risk of wire potential displacement [29].

The cost of the needle wire system is $25–30 each. The technique is a cost-effective method and does not need special training as it is already being practiced by breast radiologists and can be fairly utilized in low resource settings as well. Most commonly this technique involves placement by ultrasound guidance or mammographic or MRI and very rarely placed under CT scan guidance. The inexpensive wire localization technique has the advantage that there is widespread expertise already available, both radiologically and surgically, which helps to shorten the learning curve in placing wires in the axilla and retrieving the localized node. Limitations include logistics of timing of wire placement in the radiology suite and the operating room and the need to complete the procedure the same day. Others include patient discomfort due to the wire protruding out of the skin of the axilla, pain, hematoma and injury to adjacent soft tissue at the time of placement. With regards to the axilla, close proximity to major vessels (axillary artery and vein) and neurovascular structures adds a further safety concern and hence some institutions defer from practicing wire placement in axilla. What has been commonly seen in breast wire placement, wire migration in the axilla has also been described following arm movement or muscle contraction [30,31]. Lastly the skin entry site for the wire is often located remotely from the wire tip and target lesion or tunneled in a complex fashion, which may result in difficulty in retrieving the wire into the wound or its inadvertent migration.

### 2.3. Ultrasound Visible Clip

**Figure 3:**
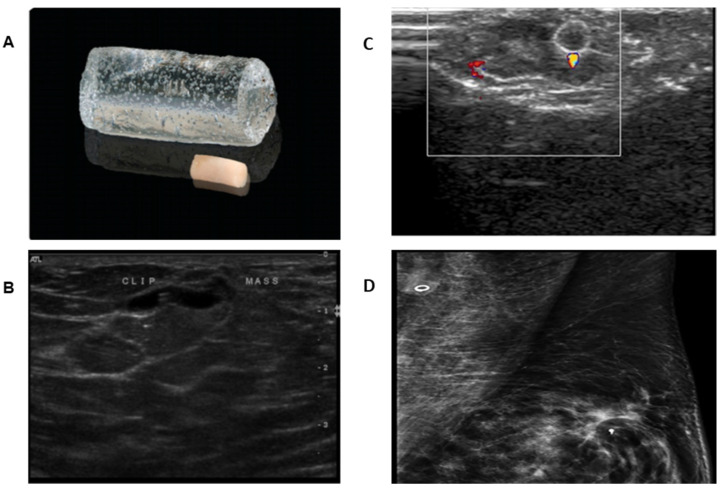
Sonographically visible HydroMARK clip. (**A**) Image showing cylinder shaped HydroMARK clip pre (small cylinder) and post (clear cylinder) absorption of water. (**B**) Transverse sonographic image of HydroMARK clip in the abnormal axillary lymph node. Typical sonographic view of HydroMark marker in the days following its implantation. From Woods et al. [33]. A review of options for localization of axillary lymph nodes in the treatment of invasive breast cancer. *Acad. Radiol*. **2019**, *26*, 805–819 (Copyright obtained from the Rightslink Copyright Clearance Center under license number 4956080252494). (**C**) Sonographically visible Twirl marker, demonstrating clip with twinkle artifact within lymph node. (**D**) Presence of Twirl clip in left axillary node on mammogram. From Tan et al. [34]. The ‘twinkle’ artifact- a novel method of clip identification to facilitate targeted axillary surgery following neoadjuvant chemotherapy in breast cancer patients. *Clin. Imaging* **2020**, *68*, 36–44. (Copyright obtained from the Rightslink Copyright Clearance Center under license number 5087861367522).

Ultrasound (US) visible markers [HydroMARK, Mammotome, Cincinnati, OH, USA) and Tumark (Hologic, Bedford, MA, USA)] consist of 3 mm metal clips (usually titanium) of three distinct shapes for better visibility contained in a bioresorbable material typically made of collagen, polyglycolic acid and polylactic acid, which is hygroscopic. These clips are deployed in the node using a vacuum assisted needle system of 8 gauge or 10 gauge needle. At first, these markers appear as an echogenic structure on ultrasound, and as they absorb water from the surrounding tissue, they expand and anchors within the target cavity. Once hygroscopic, the US visible marker appears as a hypoechoic tubular structure with a hyperechoic, double linear central artifact that corresponds to the central metal coil (Figure 3). Once deployed, the HydroMARK cylinder is not visualized on mammograms, but only the metal coil is seen but well visualized on MRI [32].

In the ILINA trial study of 46 patients which evaluated feasibility of intra operative ultrasound to detect the clipped HydroMARK nodes and removed regardless of the type of axillary surgery (SLNB or ALND), the IR was 95.7% (44/46 patients) and only two patients (4.3%) required an ALND to retrieve the clipped node [35]. The FNR was 4.1% (95% confidence interval 0.1–21.1%). According to the results obtained, IOUS-guided excision of the clipped node can be performed successfully after NAC without complications, as long as the clip is US visible prior to surgery. The authors found that there were no differences between the duration of treatment and clip visibility, but visibility of the HydroMARK clip worsens the longer it is placed in the axilla. In a study of 55 patients at the Mayo Clinic, with two types US visible markers (HydroMARK vs. Tumark) placed in the positive nodes prior to NAC and pre- and post-NAC MRI, it was found that the HydroMARK marker demonstrated three times higher likelihood of being visualized in T2-W0 images compared to Tumark (*p* = 0.003) [36]. Of the 55 markers, 40 were noted to be within the cortex while the remaining 15 were noted to be outside the cortex (within the hilum or surrounding tissues). The authors found that a marker was 1.2 times more likely to be visualized if placed in the cortex versus out of cortex but this was not statistically significant (*p* = 0.625) and they concluded that while localization of axillary LN markers after NAC is not typically performed using MRI, identification with a dedicated axillary protocol using radiofrequency coil placed on the axilla was better than a more standard protocol covering the breast and the axilla in the same field of view.

The cost of US visible hygroscopic markers is $60. Advantages are easy access to much posterior lesions, accurate placement and minimized tissue deflection. It can be sonographically visible until 12–15 months after placement thus useful for patients receiving NAC and FDA approved for long term placement in the body. It averts preoperative localization and scheduling difficulties. Being sonographically visible intraoperatively, eliminates the need for a possible specimen radiogram after removing the target LN. The hydrogel is visible till at least three months post deployment. The main disadvantage is that its visibility reduces over time due to absorption of the hydrogel fluid and difficulty detecting the echogenic titanium marker in the axilla or within the LN hilum which are both echogenic. Although the clip is small, if placed adjacent to the node, once it is hygroscopic clip migration and/or extrusion has been reported [32]. The biopsy clip is identified better when placed in the cortex of the abnormal node [37]. The UltraCor TWIRL clip is a reasonable alternative for LN marking as it consists of a larger echogenic metallic (Nitinol) component, which may be better visualized on US following NAC. The TWIRL clip comprises of two concentric rings and is more echogenic than the HydroMARK. The rings are identified rather than the two short echogenic lines of HydroMARK. However, the TWIRL clip produces more susceptibility artifacts on MRI than the HydroMARK [37]. The second disadvantage is that it requires intraoperative ultrasound which needs additional training for the surgeon and OR staff (Certification by the American Society of Breast Surgeons). The pathologist needs to be informed of the presence of HydroMARK in the LN specimen, as they tend to show a pseudo cystic lesion lined by histiocytes and foreign body reaction that can be misinterpreted as granulomatous changes, or even neoplastic cells and reported as a positive node [38].

### 2.4. Magnetic Seed Localization (MSL)

**Figure 4:**
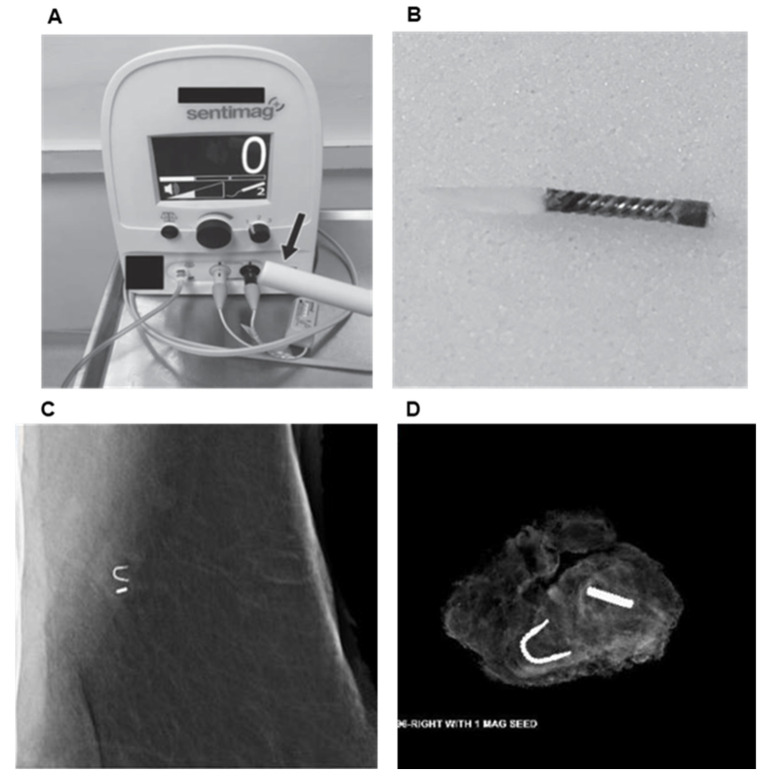
Magnetic seed localization. (**A**) Sentimag probe and console. (**B**) Magseed^®^ structure consists of 5 × 1 mm paramagnetic steel seed without the barbs of the traditional wire used for localization. (**C**) Image of Magseed^®^ deployed in axilla beside biopsy clip. (**D**) Specimen radiograph of axillary lymph node with clip and Magseed^®^. From Simons et al. [39]. Prospective Registry Trial Assessing the Use of Magnetic Seeds to Locate Clipped Nodes after Neoadjuvant Chemotherapy for Breast Cancer Patients. *Ann. Surg. Oncol.* **2021**, *28*, 4277–4283 (Copyright obtained from the Rightslink Copyright Clearance Center under license number 5131580212376).

The Magseed^®^ system (Sentimag probe, Mammotome, Cincinnati, OH, USA) was approved by the FDA for soft tissue/axilla in 2018 and localization utilizes an approximately 5 × 1 mm paramagnetic steel and iron oxide pellet (which does not have the barbs of a traditional wire used for localization), for placement in the target tissue by ultrasound or mammographic guidance. It generates an alternating magnetic field that is localized by a Sentimag probe and makes it identifiable within the target tissue. The Magseed^®^ comes in a sterile package pre-loaded in an 18gauge needle, with the seed retained in the needle by a wax plug. The seed is then deployed under ultrasound guidance, by pushing on a steel obturator, just like a marker clip. The intraoperative procedure is guided by both an audio signal and a visual counter on the console, which facilitates the removal of the target with seed (Figure 4).

An advantage is the ease of placement, as the procedure is similar to clip placement and no special training is required by the radiologists. The procedure can be done up to 30 days prior to surgery, which improves the workflow efficiency between the radiology suite and operating room. There is no signal decay over time. A limitation is cost- each seed costs $500 plus $55,000 for the probe and console. Magseed^®^ can be accurately detected at a depth of up to 4 cm but less reliably the greater the depth, which can be potentially problematic for patients with very deeply located nodes. Another major challenge is the interference of ferromagnetic instruments with the Magseed^®^ signal, hence will require use of non-ferromagnetic surgical instruments. Signals may also be interfered by electrocautery and other metallic equipment in the operating room, which may entail frequent recalibration of the probe. If the seed has been placed prior to an MRI, there may be artifacts in the images of the axilla. Concerns about non-deployment of seed or inaccurate placement are two major disadvantages of Magseed^®^. In a UCSF study by Greenwood et al. involving 35 patients with 38 seed placements (two had bilateral and one was misplaced), 86% (30/35) underwent neoadjuvant treatment with chemo/endocrine therapy, successful retrieval was documented in 97% (37/38) patients [40]. There was one reported case of seed misplacement (2.6%) from the target due to which a second seed had to be placed in the same sitting in the radiology suite and one case of failure to detect the seed in the OR/specimen radiograph and post-operative MRI and hence thought to be lost in possible local suction. No other complications were reported. In another study of 57 patients with different positive node localization methods including SAVI Scout, Magseed^®^ and RFID, failure occurred in 3/12 (25%) of patients with Magseed^®^ [41]. While the majority of failures were with Magseed^®^, the authors concluded that this likely represents the learning curve associated with targeted axillary dissection, as they trialed devices sequentially beginning with the Magseed^®^. In the largest series in United States, of 637 patients evaluating Magseed^®^ in breast and axillary lymph nodes, 136 seeds were placed in the clipped lymph nodes prior to NAC and the overall magnetic seed retrieval rate was 98.6% with a 0.7% complication rate despite the expected learning curve for a novel procedure [42]. Clinical complications included two cases of hematoma, one infection treated with antibiotics and one case of pain at deployment. More recently, in a multi institution magnetic seed study of 50 patients, all patients had successful seed placement at the first attempt with a mean time of 6.1 min for localization (range 1–30 min) and overall seed retrieval rate of 98%. No adverse events resulted from the use of magnetic seeds and only 4% surgeons rated the procedure as difficult during their first three cases suggesting a learning curve [39]. The authors concluded that magnetic seeds allow for the convenience of seed localization without the regulatory burden associated with radioactive seeds. Disadvantages associated with magnetic seed placement in the axilla before MRI include susceptibility artifacts from the seed which may obscure axillary details; while intraop disadvantage includes use of plastic instruments to avoid signal interference, which are cumbersome in the axilla. Presently a large trial is underway at the MD Anderson Cancer center to study the efficacy of Magseed^®^ and Sentimag to localize axillary lymph nodes following NAC (https://clinicaltrials.gov/ct2/show/NCT03796559, accessed date 20 October 2020) [43].

### 2.5. I125 Radioactive Seed Localization (RSL)

**Figure 5:**
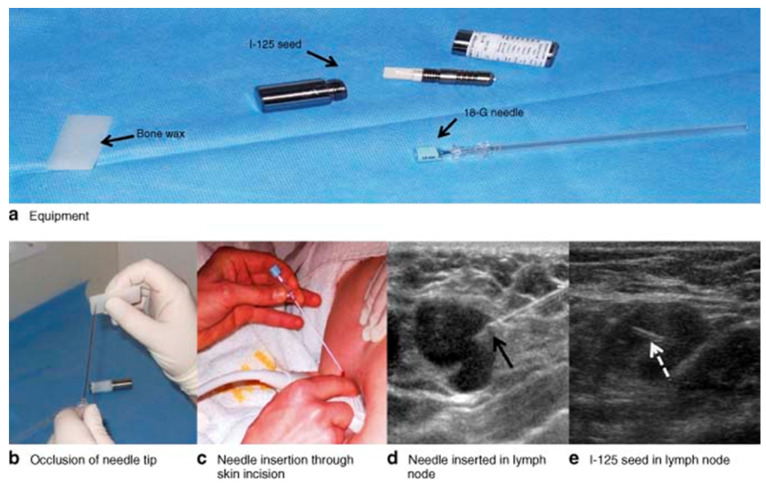
Radioactive I-125 seed placement. Positioning of an iodine-125-radiolabelled (I-125) seed in an axillary lymph node. (**a**) Equipment. (**b**) The tip of an 18-G needle is occluded with sterile bone wax and the I-125 seed is placed in the needle. (**c**) The needle is inserted through a skin incision. (**d**) The lymph node (hypoechoic) is visualized by ultrasonography and the tip of the needle (arrow) is inserted in the lymph node. (**e**) The I-125 seed (arrow) is moved through the bone wax and into the lymph node using a stylet. From Straver et al. [44]. Marking the axilla with radioactive iodine seeds (MARI procedure) may reduce the need for axillary dissection after neoadjuvant chemotherapy for breast cancer. *Br. J. Surg.* **2010**, *97*, 1226–1231 (Copyright obtained from Rightslink Copyright Clearance Center under license number 5022600645561).

This procedure involves placement of a titanium encapsulated ^125^I seed (STM1251, Bard Brachytherapy Inc., Carol Stream, IL, USA) with an average energy of 27 keV and a half-life time of 59.6 days in an 18-gauge needle. The radioactivity level in the seed ranges from 0.01 to 0.3 millicuries (mCi) and has been considered safe for human exposure by the Nuclear Regulatory Commission (NRC). Under ultrasound guidance, the ^125^I seed is placed in a proven metastatic or clipped axillary lymph node and a local x-ray is used to confirm the position of the seed (Figure 5). Radiation safety protocols and detailed documentation regarding the acquisition, handling, and storage including guidelines for patients and hospital staff should be maintained.

The MARI (marking the axilla with radioactive iodine seeds) procedure was first described in the axilla by Straver et al. in 2010 [44]. In a pilot study of 15 patients, it was proven to be a feasibility study for evaluation of tumor positive axillary lymph nodes marked with ^125^I and selectively remove them following NAC. With the γ -probe (neo2000, Neoprobe Corporation, Dublin, OH, USA) on the ^125^I setting, the point of greatest activity was detected on the skin of the axilla. The incision to remove the MARI node was made in the planned incision for the ALND, close to the point of highest activity. Guided by the γ -probe, the MARI nodes were intraoperatively detected and selectively removed. Removal of the correct lymph node was ensured by detecting the ^125^I source of radioactivity within the lymph node ex vivo and by the absence of radiation in the rest of the axilla. In this pilot study, the successful placement of the seeds in the LN was 100%, with no complications and a median time to definitive surgery of 123 days (range 88–193 days). As a follow up to the above study, the same group conducted the MARI procedure in 103 patients with positive ALN for definitive axillary surgery after NAC. They found a high identification rate of 97% and low FNR 7% [45]. They concluded that MARI procedure is a safe technique and easy to learn, but with radiological insertion challenge if the node is small, and, therefore, confirmation of the localization with ultrasound is necessary. Surgical removal of the MARI node requires skills comparable with the removal of an SN. The Radioactive Iodine Seed Localization in the Axilla with Sentinel Node Procedure (RISAS) study has enrolled 225 cN+ patients who were subjected to NAC and undergo MARI as well as SLNB [46]. The primary endpoint was accuracy of the RISAS procedure. The identification rate, false-negative rate, negative predictive value, and possible concordance between the MARI and SLNB will be reported. Results of this trial are pending. In the first series of ^125^I seed placement in clipped nodes following NAC, Caudle et al. observed that the IR of the clipped node with ^125^I seed was 100% and adding evaluation of the clipped node to evaluation of the SLNs reduced the FNR to 1.4% (95% CI, 0.03 to 7.3) from the FNR of 10.1% for SLND alone. However, in comparison to the MARI procedure that involved marking the clipped node with ^125^I before starting the NAC, the authors suggested that leaving the radioactive seed in place throughout chemotherapy is not in line with current US regulations [47].

One study from the Mayo Clinic has reported CT-guided localization of radioactive seed in both non-palpable breast and positive axillary nodes. For axillary lesions the greatest thickness of the cortex or the largest diameter was noted. In the seven axillary lesions with positive nodes with clip, an 18G coaxial needle system with a preloaded ^125^I radioactive seed was inserted on the day of or 1–5 days before surgery. IR was 100% with no complications related to bleeding, infection or vasovagal response [48]. The only minor complication reported was slight seed displacement of 2–8 mm from three (30%) of the targeted axillary nodes and clips and on pathology, the seed was noted to be outside of the node. All radioactive seeds and targeted lesions were successfully removed during surgery. More recently, Custodio et al. evaluated the safety of placement of ^125^I radioactive seeds in patients with stage II-III breast cancer and found the IR to be 100% with no complications [49].

Cost of the seed is $17 to $60 per seed, $15,000 for probe and $30,000 for the console. Advantages are that the procedure of seed placement can be done days to months prior to the definitive axillary surgical procedure and thus improves workflow efficiency. It does not require extra training as the procedure is similar to placing a clip. the identification of the affected lymph node is independent of changes in lymphatic flow due to treatment. The downside of the procedure is that it requires good understanding of the handling of radioactive material by the personnel. It also requires proper collection and counting of the seed from the OR specimen and disposal according to the regulatory guidelines. When the seed is retrieved, it should be immediately placed in a specimen container, which should be labeled with patient identifiers and a “Caution—Radioactive Materials” label and placed in a shielded container. The recovered seed must be transferred to the institution’s nuclear medicine or radiation safety department for appropriate disposal. The extrusion rate of the seeds is still largely unknown.

### 2.6. SAVI SCOUT

**Figure 6:**
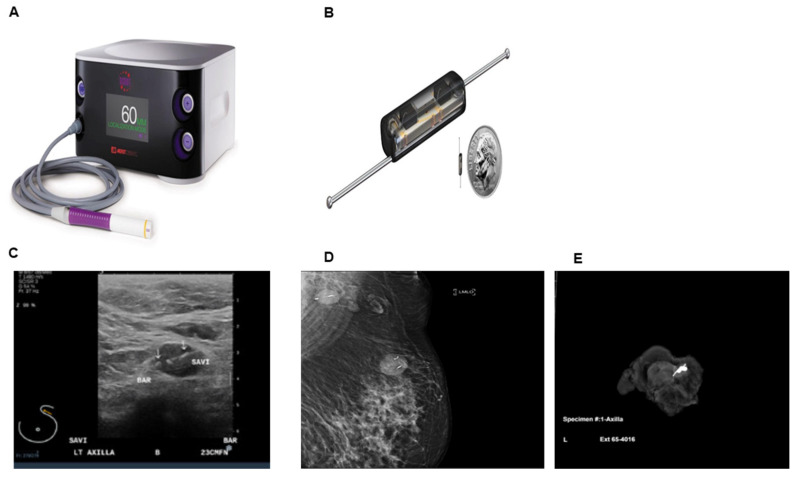
Radar and infrared light system SAVI SCOUT (Merit Medical). (**A**) SAVI SCOUT Console with handheld probe. (**B**) SAVI SCOUT reflector size comparison with a dime (**C**) Image of ultrasound guided placement of reflector in the axillary lymph node with clip (**D**) Post placement mammogram demonstrates reflector in the axillary lymph node and breast (**E**) Specimen radiograph of axillary lymph node with clip and reflector. Images courtesy of Merit Medical, South Jordan, UT, USA).

SAVI SCOUT (Merit Medical Inc, South Jordan, UT, USA) is a non- radioactive wireless localization technique which uses a combination of micro impulse radar and infrared light from a handheld detector that enables as a localization method. It received FDA approval for placement in breast lesions in 2014 and for long term placement in soft tissue/axilla in 2018, with no restrictions in length of time the reflector can remain in the breast. The localization system consists of an implantable reflector, a handpiece and a console. The reflector is 12 mm long and consists of 4 mm body and two 4 mm nitinol antennae and is percutaneously placed by ultrasound or mammographic guidance via a 16-gauge needle in a preloaded delivery system. The reflector consists of an IR light receptor, resistor switch and two antennae which is placed into or near the target through a 16 G needle under mammographic or sonographic guidance. The handpiece and console system emit pulses of infrared (IR) light and radar wave signals and receives signals back from the reflector to provide real-time localization and target proximity information to the surgeon (Figure 6) [50]. SCOUT has been used in lymphoma (nodes), melanoma (nodes and site metastasis), soft tissue sarcoma and lung. While most published reports are based on reflectors placed in the non-palpable breast lesions, only one report has published its utility in the axillary nodes. In a study of 45 patients who cN+ and biopsy proven metastatic lymph node who received NAC, SCOUT was placed in all patients with a median of 8 days (range 1–167) prior to surgery. The procedure was safe with no perioperative or post op complications, with IR of 100% and 65% demonstrated pathologic treatment effect [51]. Seven out of 45 patients (15.5%) had the reflector placement done at the time of initial positive biopsy, which shows promise of reducing the number of invasive procedures for patients after NAC.

The cost of single use reflector is $400, and the console and probe are $40,000. The major advantage of this device is the ability for long-term placement before surgery thus improving workflow efficiency. SCOUT can be placed up to 30 days prior to the definitive surgery. It can be easily identified by pathologists in the surgical specimen. Combination of the auditory cue and reading on the LCD display indicating the proximity to the tag enhances the surgeon’s subjective ability to localize the reflector and hence the lesion. There is no radioactivity involved and hence potentially saves the patient and personnel of any radioactive exposure. There is no reported reflector migration or patient discomfort with arm movement reported. Since the technology is very accurate, it helps in precise surgical planning of the axilla and retrieval of the nodes. The clip is MRI compatible with less MRI bloom than other commercially available implantable localization devices, which allows marked lesions to be evaluated in the course of treatment [52]. However, it has some disadvantages. The size of the reflector is 12 mm and hence may be at times bigger than the node itself and may have to be placed outside the cortex of the targeted node. Interference with older halogen lights in the OR has been reported, because older technology halogen lights emit infrared radiation which could impact reflector detection. It is relatively more expensive than the other methods of localization [52]. In a study of 123 reflector placements in breast lesions of 100 women, only one case of hematoma in the axilla and hence reflector migration has been reported thus far [53].

## 3. Conclusions and Future Directions

Marking the biopsy proven metastatic axillary LN at the time of core biopsy is important for its identification at the time of definitive axillary surgery, as this significantly improves the ability to remove the known positive node. Given the critical role of assessing the previously positive node to confirm pCR after NAC and performing axillary surgery with minimal morbidity, retrieval of this previously marked clipped node is key. We understand the notion that identification of metastatic lymph nodes after NAC may not be critical if residual cancer cells are to be killed by adjuvant regional radiation or systemic therapy. On the other hand, we argue that those residual cancer cells may be more resistant to adjuvant chemotherapy or radiation because they already survived NAC. We believe that the clinical relevance of residual lymph nodes will be clarified by a trial that specifically addresses removing those nodes. In this review, we summarized the techniques used to mark those nodes. Despite a wide spectrum of options available for localizing the clipped node, there is insufficient evidence to support any one specific technique. The choices depend on the node location, experience of the radiologist and the surgeon, and logistics such as widespread availability and cost. Newer techniques being presently employed are the Envisio (Lucid system) which uses electrocautery pen and electromagnetic clips that can be placed for up to 1year prior to surgery. Other newer technologies include Magseed biopsy with Magseed PRO uses a smaller seed and avoids MRI artifacts and Localizer (Hologic, Bedford, MA, USA) which is an advanced RFID tag that uses a mobile handheld reader and is not dependent on a console connected with a wire placed at a distance. Magtrace is a liquid used for SLNB which can be injected intra or peritumorally in even premalignant and DCIS disease where the axilla may be addressed later, if the pathology comes back with microinvasion. The tracer is also dark in color that offers the surgeon a visual confirmation and helps eliminate the need for blue dyes. These newer techniques promise precision and effective mechanisms for both deployment and retrieval with no interaction with imaging artifacts or interaction with electrocautery, but need larger studies to evaluate their efficacy. With more breast cancer patients being subjected to NAC, further studies will be required to determine the optimal method of clipped node localization which can be safe, cost effective, approves patient satisfaction, workflow conducive and easily retrievable, and practice it as a standard of care. Data of longer follow-up must determine whether implementation of this protocol is untimely and whether it not only reduces axillary morbidity but also preserves oncologic safety in terms of disease-free and overall survival. Prospective trials with sufficient follow-up are therefore needed.

## Figures and Tables

**Table 1:** False negative rates in patients subjected to Sentinel lymph node biopsy following neoadjuvant therapy.

Trial Name [Ref. Number]	False Negative Rate (%)
SENTINA [9]	14.2%
SN-FNAC [10]	8.4%
Z1071 [7]	12.6%
GANEA2 [8]	11.9%

**Table 2:** Comparison of different techniques of axillary lymph node localization with cost analysis, benefits and pitfalls.

Procedure	Cost (US$)	Advantages	Disadvantages
Carbon suspension/Charcoal tattoo	$100 per 5 mL vial	100% identification in node Seen up to 6–8 months after injection No side effects	Color misidentification Possibility of some ink migration to non-tattooed node or surrounding tissue One report of foreign body granulomas
Wire Localization	$25–30 per wire	Cost effective	Same day as surgery Pt discomfort, pain, hematoma, adjacent tissue injury High chance of migration due to arm movement, muscle contraction
Ultrasound visible clip	$60	Small in size 3 mm Sonographically visible up to 12–15 months Newer ones UltraCor, O-Twist not water absorbing	Will need intraop ultrasound to detect the clip Needs instrument confidence by the surgeon Hygroscopic, absorbs water and subject to migration Decreased visibility over time
Magnetic seed localization	$500 per seed $55,000 for probe and Console	Can be placed up to 30 days prior to surgery	Depth limitation 3.5 cm Cost Calibration required and susceptible to other magnetic fields If done prior to MRI breast, artifact in the axilla
I 125 radioactive seed localization	$17–60 per seed $15,000 for probe $30,000 for Console	Small size 4 × 0.8 mm Long half-life (60 days) Detectable up to 4 months post NAC by Gamma probe	Strict Nuclear Regulatory Commission protocol →Complete chain important from insertion to removal to retrieval of all seeds Migration chances more intraop than preop
SAVI SCOUT	$400 for Single use reflector $40,000 for probe	Up to 6–8 cm depth detection Operating room efficiency Long term placement up to 30 days before surgery	One report of migration due to hematoma
